# Thymic stromal lymphopoietin in hepatitis C virus-related cryoglobulinemic vasculitis: gene expression level and protein distribution

**DOI:** 10.1186/s13075-015-0581-x

**Published:** 2015-03-15

**Authors:** Domenico Sansonno, Sabino Russi, Silvia Sansonno, Fabio Pavone, Franco Dammacco

**Affiliations:** Liver Unit, Department of Biomedical Sciences and Human Oncology, University of Bari Medical School, 11 Piazza G. Cesare, 70124 Bari, Italy; Institute of Internal Medicine, Department of Medical and Surgical Sciences, University of Foggia Medical School, 1 viale L. Pinto, 71122 Foggia, Italy

## Abstract

**Introduction:**

Hepatitis C virus (HCV) infection can be detected in virtually all patients with cryoglobulinemic vasculitis (CV). Among its many effects, the virus is able to stimulate the production of thymic stromal lymphopoietin (TSLP) by infected hepatocytes. In this study, we assessed the systemic levels and tissue distribution of TSLP in 60 chronically HCV-infected patients, 36 with and 24 without CV.

**Methods:**

Serum TSLP levels were measured by an enzyme-linked immunosorbent assay (ELISA) method. TSLP mRNA was assessed in patient samples by real-time reverse transcriptase-polymerase chain reaction (RT-PCR). TSLP protein in liver and skin biopsy samples was revealed by indirect immunofluorescence. All other methods were carried out according to standardized procedures.

**Results:**

Serum TSLP levels were significantly higher in patients with than in those without CV and in healthy individuals. Higher TSLP levels paralleled specific mRNA expression and the up-regulation of TSLP protein in liver tissue. Compared with non-CV patients, higher TSLP levels in CV were accompanied by a higher frequency of circulating mono/oligoclonal B-cell expansions (8% vs. 92%, p < 0.0001) and a higher number of peripheral CD20^+^ B-cells (10.3% vs. 15.5% p = 0.04). In addition, TSLP mRNA expression in the liver of CV patients was lower than in their correspondent skin tissue and paralleled specific immune deposits of TSLP protein in keratinocytes.

**Conclusion:**

Overall, this study shows that TSLP secreted by hepatocytes and keratinocytes of HCV-infected patients with CV is involved in the pathogenesis of vasculitis and may possibly support the therapeutic use of TSLP-targeted monoclonal antibodies.

## Introduction

Thymic stromal lymphopoietin (TSLP) is a four-helix-bundle cytokine and a member of the common γ-chain cytokines, which are able to induce dendritic cells (DCs) and to stimulate naïve T-cell differentiation into T-helper 2 [1] and T-helper 17 [2] cells. TSLP binding and signaling occur by means of a heterodimer composed of the interleukin-7 receptor α-chain and the TSLP receptor [3].

TSLP is a potent modulator of systemic B-cell development and is capable of promoting humoral autoimmunity. In the skin of a genetically engineered mouse, TSLP released into the systemic circulation by Notch-deficient keratinocytes induced a remarkable expansion of peripheral pre-B cells and immature B lymphocytes, resulting in B-lymphoproliferative disorders and death [4]. In addition, local expression of TSLP under the control of a tetracycline-regulated, skin-specific promoter caused a substantial increase in bone marrow B lymphocytes and an earlier exodus of immature cells to the periphery [5]. These changes led to an increase in antibody-secreting cells, the production of mixed cryoglobulins, immune-complex-mediated renal damage [6], and systemic inflammatory injury, an overall picture closely resembling human cryoglobulinemic vasculitis (CV) [7].

In the Mediterranean basin, over 90% of CV patients are chronically infected with hepatitis C virus (HCV), thus emphasizing the role of this virus in the pathogenesis of cryoglobulin production. However, only a subset of HCV-positive individuals develops mixed cryoglobulins and only a minority of these patients has clinically overt CV [8]. B-cell clonal expansions in the circulation and in the liver microenvironment are peculiar features of the humoral immune response of CV patients [9]. In addition, dominant B-cell clonalities probably contribute to the formation of intraportal follicle-like structures in the liver [10]. Analysis of the immunoglobulin heavy chain complementarity-determining region CDR-3, whether from circulating or tissue-derived B-cell-expanded clones, showed several variations in this immunoglobulin gene segment, supporting the notion that these cells are the result of an antigen-driven response [11]. Restriction in the use of the B-cell V gene was shown to have a direct clinical impact in CV patients, based on its association with higher levels of rheumatoid factor activity and with lymphoproliferative disorders [12,13].

Recently, it has been reported that the infection of hepatocytes *in vitro* by HCV results in a remarkable production of TSLP [14] through a mechanism regulated in a nuclear factor-κB-dependent fashion, and that TSLP is able to enhance the release of T-helper 17 differentiating cytokines by DCs. In view of this finding, it can be argued that upregulation of hepatocyte-derived TSLP plays a major role in the loss of B-cell tolerance, resulting in the drastic expansion of B-cell populations and the stimulation of cryoglobulin production in chronically HCV-infected patients. Since TSLP is required for the initial expansion of B1 and B2 bone marrow B-cell progenitors [15], it can also be postulated that an increase in systemic TSLP levels in HCV-infected patients enhances B-cell lymphopoiesis and the expansion of specific B-cell subsets, leading to override of some of the controls underlying B-cell tolerance.

Here, we asked whether an *in vivo* inducible upregulation of TSLP can be shown in patients with chronic HCV infection and CV. A possible relationship between TSLP and HCV nucleocapsid core protein, devoid of envelope proteins, as a constitutive component of cryoglobulins and potentially able to cause cryoglobulin-mediated tissue injury [16] was also investigated. Our data indicate that high serum levels of TSLP parallel those of specific mRNA transcripts, both in the liver and to a higher extent in the skin of HCV-infected patients, suggesting that this cytokine plays an important role in the pathogenesis of CV-related tissue damage.

## Materials and methods

### Patients and controls

Thirty-six naïve patients with a diagnosis of CV and the classical symptom triad of palpable purpura, arthralgia, and asthenia were enrolled in this study. Eligibility criteria were as follows: no previous therapy with interferon, steroids, or immunosuppressive drugs; serum positivity for anti-HCV antibodies and HCV RNA; liver histology compatible with chronic active hepatitis; negativity for serum HBsAg and anti-HIV antibodies; alcohol intake <40 g/day; and currently not pregnant. The study was approved by the Independent Ethical Committee (Azienda Ospedaliero-Universitaria ‘Policlinico Consorziale’, Bari, Italy); written, informed consent was obtained from all patients, in accordance with the declaration of Helsinki.

Serum cryoglobulins were isolated and characterized as described elsewhere [17]. In addition to diagnostic liver biopsy, all HCV-infected patients underwent two 3 mm punch biopsies from two cutaneous lesions. Control skin samples were derived from 24 chronically HCV-infected patients (16 males, mean age: 55.2 ± 14.9 years) who tested negative for mixed cryoglobulinemia on three or more occasions and from 10 healthy subjects (six males, mean age: 48 ± 9.4 years), all of them undergoing surgical procedures because of inguinal or abdominal hernias, subcutaneous nodules, cutaneous squamous cell carcinoma, saphenectomy, or nodal nevi. Liver and skin biopsy specimens were either formalin-fixed and paraffin-embedded for routine histological examination or embedded at the optimal cutting temperature, snap frozen, and stored at −80°C until sectioning for use in immunofluorescence studies. Portions of the biopsy samples were prepared for molecular analyses. The specimens were placed in RNase-free microtubes and immediately frozen in liquid nitrogen until RNA extraction.

Patients were treated with pegylated interferon alpha/ribavirin (pIFNα/RBV) combination therapy. Those infected with genotype 2 and genotype 3 were treated for 6 months, and those infected with genotype 1 for 12 months. Patients were reassessed 6 months after discontinuation of therapy to establish whether they had achieved a sustained virological response.

Virological, epidemiological, histological, and laboratory features as well as major symptomatology are summarized in Table 1.

**Table 1:** **Virological, epidemiological, histological, laboratory, and clinical characteristics of chronically HCV-infected patients with and without cryoglobulinemic vasculitis**

	**With CV (** ***n*** **= 36)**	**Without CV (** ***n*** **= 24)**	***P*** **value** ^**a**^
Virology			
Serum HCV RNA	36 (100)	24 (100)	
• Titer (log IU/ml)	5.9 (4.3 to 6.9)	6.2 (5.9 to 6.7)	NS
Serum HCV core protein	36 (100)	24 (100)	
• Titer (pg/ml)	15 (0.8 to 130)	46 (0.2 to 385)	0.08
HCV genotype			
• Genotype 1	21 (58)	14 (58)	NS
• Genotype 2	15 (42)	8 (33)	NS
• Genotype 3	0	2 (9)	NS
Anti-HCV antibodies	36 (100)	24 (100)	
HBsAg	0	0	
Anti-HIV antibodies	0	0	
Epidemiology			
Sex: male/female (ratio)	9/27 (0.33)	16/8 (2)	0.003
Age (years)	58.3 ± 9.5	55.2 ± 14.9	NS
Blood/blood product transfusion	12 (33)	9 (37.5)	NS
Liver histology (METAVIR scoring system)			
F0	2 (5.6)	0	NS
F1	6 (16.7)	4 (16.7)	NS
F2	11 (30.5)	9 (37.5)	NS
F3	8 (22.2)	4 (16.7)	NS
F4	9 (25)	7 (29.1)	NS
Laboratory			
Cryocrit (%)	4 (1 to 34)	0	
Immunochemical type			
II	33 (91.7)		
III	3 (8.3)		
RF (IU/ml, ≤20)	101 (2 to 2,950)	12 (2 to 28)	<0.0001
Immunoglobulins			
IgM (mg/dl, 40 to 230)	294 (73 to 1,050)	142 (28 to 201)	<0.0001
Complement fractions			
C1q (mg/dl, 21 to 39)	37 (34 to 75)	28 (23 to 46)	NS
C3 (mg/dl, 90 to 180)	102 (44 to 167)	101 (85 to 109)	NS
C4 (mg/dl, 10 to 40)	2.5 (1 to 19)	14 (9 to 20)	<0.0001
ALT (IU/l, ≤30)	59.5 (33 to 124)	83 (63 to 231)	NS
Peripheral lymphocytogram			
CD20 (%, 10.2 ± 5.4)	15.5 (5.5 to 81)	10.3 (7 to 25)	0.04
Circulating B-cell clonalities			
Monoclonal	21 (58.4)	0	<0.0001
Oligoclonal	12 (33.3)	2 (8.3)	0.03
Polyclonal	3 (8.3)	22 (91.7)	<0.0001
Clinical features			
Palpable purpura	27 (75)	0	
Weakness	24 (66.7)	4 (16.7)	
Arthralgias/nonerosive arthritis	27 (75)	3 (12.5)	
Cutaneous ulcers	21 (58.3)	1 (4.2)	
Peripheral neuropathies	18 (50)	0	
Renal disease	9 (25)	0	

Data presented as *n* (%), median (range) or mean ± standard deviation. CV, cryoglobulinemic vasculitis; HCV, hepatitis C virus; NS, not significant; RF, rheumatoid factor. ^a^ *P* <0.05 was defined as significant.

### Laboratory parameters

Serum HCV antibodies were detected by second-generation enzyme-linked immunosorbent assay and then confirmed by third-generation enzyme-linked immunosorbent assay (Abbott Lab, Chicago, IL, USA) in the first 18 patients enrolled in this study. The last method was consistently employed in the remaining 42 patients. Serum HCV RNA was determined by RT-PCR (Roche Diagnostics, Branchburg, NJ, USA) and quantified with the Versant HCV RNA quantitative 3.0 assay (Siemens Healthcare, Erlangen, Germany). HCV genotyping was performed with INNO-LiPA (Innogenetics NV, Ghent, Belgium).

### Soluble thymic stromal lymphopoietin

Immunoreactive TSLP in serum samples was quantified using an enzyme-linked immunosorbent assay with matched antibodies, according to the basic laboratory protocol provided by the manufacturer (R&D Systems, Minneapolis, MN, USA). TSLP protein was quantified with reference to serial dilutions of the recombinant standards, falling within the linear part of the standard curve for each specific TSLP value measured. The sensitivity threshold of the TSLP assay is 6.7 pg/ml. Each data point represents readings from two independent assays performed in duplicate.

### Circulating hepatitis C virus core protein

Serum nonenveloped HCV core protein was tested using Architect HCV Ag (Abbott Diagnostics, Wiesbaden, Germany), a fully automated, quantitative, chemiluminescent microparticle immunoassay for the detection of nonenveloped HCV core protein in HCV-infected sera [16].

### Tissue RNA isolation and PCR amplification

Total RNA was extracted from liver and skin samples with the RNeasy Mini kit (Qiagen, Hilden, Germany), according to the manufacturer’s protocol, and reverse transcribed to cDNA using the iScript Select cDNA synthesis kit (Bio-Rad Laboratories, Hercules, CA, USA). Five microliters of cDNA were used in a nested PCR for HCV RNA detection together with the AmpliTaq Gold PCR master mix (Applied Biosystems, Foster City, CA, USA) and the following primer sets targeting the 5′-untranslated region: sense 5′-GGGGGCGACACTCCACCA-3′ (position 15 to 32) and anti-sense 5′-TCGCGACCCAACACTACTC-3′ (position 256 to 274) for the first round of amplification; and the inner primers sense 5′-GAGTGTCGTGCAGCCTCCAG-3′ (position 98 to 117) and anti-sense 5′-CTCGGCTAGCAGTCTCGCGG-3′ (position 239 to 258) for the second round.

An additional PCR for TSLP mRNA was carried out using 5 μl cDNA, the AmpliTaq Gold PCR master mix (Applied Biosystems), and the QuantiTect human TSLP primer assay (Qiagen). This primer set targets exons 1/2/3 and amplifies a region of 94 base pairs in the two transcripts (2,629 base pairs and 3,834 base pairs, respectively).

### Real-time RT-PCR for TSLP mRNA quantification

TSLP mRNA was measured in patient samples by absolute real-time RT-PCR quantification. The reaction was performed using the QuantiTect human TSLP primer assay (Qiagen) and FastStart DNA Master SYBR Green I (Roche Diagnostics, Mannheim, Germany) on a LightCycler 1.5 instrument (Roche Diagnostics), according to the manufacturer’s protocol. The cycling conditions were 95°C for 15 minutes, 45 cycles of 94°C for 15 seconds, 55°C for 20 seconds, and 72°C for 20 seconds. The final concentration of TSLP mRNA was expressed as copies of mRNA per nanogram of total RNA.

Real-time RT-PCR for HCV RNA quantitation was performed using the RNA Master HybProbe kit (Roche Diagnostics) and the following primers and probes: 5′-AGCGTCTAGCCATGGCGT (sense), 5′-CAAGCACCCTATCAGGCAGT (antisense), 5′-LC640-CCCGGGAGAGCCATAGTGGTCTG--PH (5′LCRed640) and 5′-GCAGCCTCCAGGACCCCCC--FL (3′HCV-G-Fitch) (TIB MOLBIOL, Berlin, Germany). To generate a standard curve, serial dilutions of the AcroMetrix HCV Mid Control (AcroMetrix, Benicia, CA, USA) were prepared. The HCV RNA concentration was calculated as International Units per nanogram of total RNA; the sensitivity was 20 IU. Each sample and standard curve point was run in duplicate.

### Construction of standards for real-time RT-PCR absolute quantification

The TSLP standard was constructed by cloning product of real-time RT-PCR obtained using a bioinformatically verified primer assay (QuantiTect Primer Assay; Qiagen). The PCR product was visualized on a 2% agarose gel and the respective band was excised and loaded onto a DNA purification column (DNA gel extraction kit; Millipore, Billerica, MA, USA). The purified PCR product was cloned into a pGEM-T Easy vector using pGEM-T Easy vector system II (Promega, Madison, WI, USA), according to the manufacturer’s protocol. The recombinant vector was transformed into JM109 *Escherichia coli* competent cells (Promega). The transformants were plated onto duplicate LB/ampicillin/IPTG/X-Gal plates, incubated overnight at 37°C, and then selected and processed for plasmid isolation. Five milliliters of Luria-Bertani broth cultures of single colonies were grown overnight at 37°C with shaking at 200 rpm. The DNA plasmid was purified using the QIAprep spin miniprep kit (Qiagen) and then solubilized in 100 μl of 10 mM Tris–HCl (pH 8.5) buffer. To confirm the sequence of the cloned product, DNA sequencing was performed using the BigDye Terminator v1.1 cycle sequencing kit and ABI Prism 310 genetic analyzer (both Applied Biosystems). The purified plasmid was quantified and the copy number calculated as follows:

$$ \begin{array}{l}\mathrm{Number}\kern0.5em \mathrm{of}\kern0.5em \mathrm{copies}/\upmu \mathrm{l}=\frac{6.023\times {10}^{23}\left(\mathrm{molecules}/\mathrm{mole}\right)\times \mathrm{pDNA}\kern0.5em \mathrm{concetration}\kern0.5em \left(\mathrm{g}/\upmu \mathrm{l}\right)}{\left[\mathrm{pGEMT}\hbox{-} \mathrm{T}\kern0.5em \mathrm{length}+\mathrm{TSLP}\kern0.5em \mathrm{length}\right]\kern0.5em \left(\mathrm{bp}\right)\times 660\kern0.5em \left(\mathrm{g}/\mathrm{mole}\right)}\\ {}6.023\times {10}^{23}\left( molecules/ mole=\kern0.5em  Avogadro\hbox{'}s\kern0.5em  number\right)\\ {}660\kern0.5em \left(g/ mole\right)= Average\kern0.5em  weight\kern0.5em  of\kern0.5em a\kern0.5em  single\kern0.5em  base\kern0.5em  pair\end{array} $$

where 6,023 × 10^23^ (molecules/mole) is Avogadro’s number and 660 (g/mole) is the average weight of a single base pair. The standard curve was prepared from five-point serial 10-fold dilutions, starting from 300,000 copies.

### Thymic stromal lymphopoietin protein detection

TSLP protein in liver and skin biopsy samples was detected by indirect immunofluorescence, using a goat anti-human TSLP antibody (Biorbyrt, Cambridge, UK) at a working concentration of 10 μg/ml. As a positive control, human tonsil sections were employed. The samples were incubated with the primary antibody for 3 hours at room temperature. Isotype-matched antibodies were used for control staining and a fluorescein isothiocyanate-conjugated rabbit anti-goat antibody F(ab)_2_ fragment (Dako Denmark, Glostrup, Denmark) as the secondary reagent. The latter was incubated with the samples for 2 hours at room temperature. To block a positive reaction on selected samples, the primary antibody was preincubated with human recombinant TSLP protein (5 ng/ml).

### Hepatitis C virus core protein detection

A two-stage indirect immunofluorescence procedure was carried out as described previously [18], using an antibody (clone 4,6 E7-F6) that recognizes amino acids 29 to 43 (working concentration 0.9 pg/ml).

### B-cell clonal expansions

The immunoglobulin heavy chain variable-diversity-joining gene segments were amplified in duplicate reactions according to the Fr3 protocol, as described elsewhere [9,10]. Consensus primers for the V and J regions were: an upstream primer complementary to the third framework V region, 5′-ACACGGC(C/T)(C/G)TGTATTACTGT-3′; and a downstream primer directed to a conserved sequence of the J region, 5′-TGAGGAGACGGTGACC-3′, and in the second-round amplification of an inner conserved sequence of the same J region, 5′-GTGACCAGGGTNCCTTGGCCCCAG-3′.

### Statistical analysis

Descriptive statistics included the mean or median as appropriate for continuous variables, and the frequency (%) for categorical variables. In the univariate analysis, chi-square and Fisher’s exact tests were used as appropriate to compare categorical variables, and the nonparametric Mann–Whitney test to compare continuous variables. The differences were considered significant at *P* <0.05. A Spearman rank correlation coefficient (*r*) test was used to evaluate the relationships between variables. All statistical analyses were performed using IBM SPSS statistics version 19.0 (Armonk, NY, USA).

## Results

### Study population

As shown in Table 1, all CV patients were anti-HCV-positive, viremic, and had circulating nonenveloped HCV core protein. Except for a moderate prevalence of genotype 1, no distinct distribution profile emerged among the patients. Liver damage was histologically defined. Laboratory parameters indicated normal serum levels of complement components C1q and C3, but remarkably low serum levels of C4. Molecular analyses aimed at determining immunoglobulin heavy chain variable-diversity-joining gene rearrangements showed that B-cell clonalities were expanded in 33 (92%) CV patients and in only two (8%) non-CV patients (*P* <0.0001). The mean percentage of CD20^+^ B cells was higher in CV than in non-CV patients (*P* = 0.04).

There was a strikingly higher number of female patients with mixed cryoglobulinemia (27 out of 36, 75%). This reflects the higher female prevalence in all major cohorts of CV – including our own series of 141 patients, 68% of whom were females [19]. However, serum TSLP levels were not significantly different between sexes in each group of patients.

Among the clinical symptoms of CV patients, palpable purpura, asthenia, and arthralgia/arthritis were recorded with high frequency. Peri-malleolar and/or pre-tibial cutaneous ulcers were also found in over one-half of the patients. Long-lasting hyperpigmentation affecting the legs to a variable extent, as the result of recurrent purpuric eruptions, was also almost invariably present. Skin histology showed leukocytoclastic vasculitis in 32 patients (89%), whereas pandermal vasculitis characterized by vessel thrombosis and severe endothelial alterations was diagnosed in the remaining four patients (11%). Sensory-motor peripheral neuropathy was demonstrated in one-half of the patients, in whom electromyography prevalently revealed symmetrical, distal polyneuropathy and, less frequently, mononeuritis multiplex. Renal damage was diagnosed in 25% of the patients, with renal biopsy showing membrano-proliferative glomerulonephritis in 78% and membranous nephropathy with segmental glomerulosclerosis in the remaining 22%.

### *In situ* TSLP and HCV core protein immune detection

**Figure 1:**
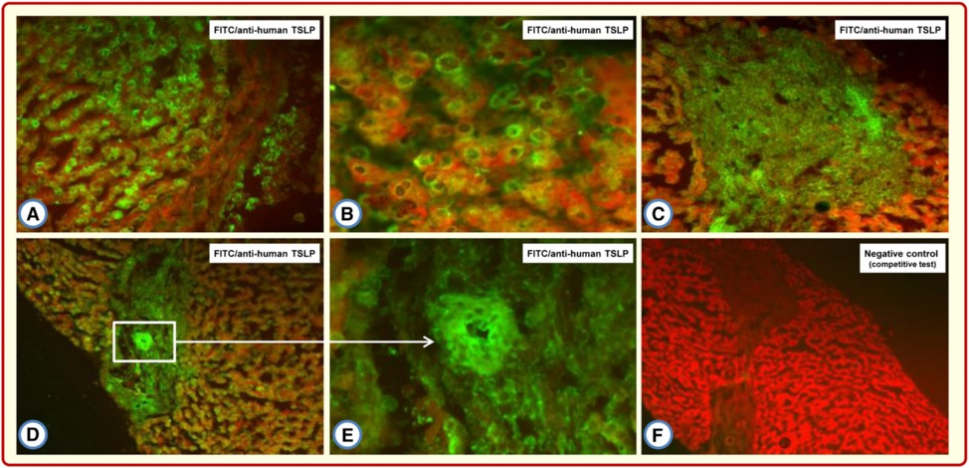
**Representative features of thymic stromal lymphopoietin immunodetection in liver biopsy specimens from patients with cryoglobulinemic vasculitis.** Specific fluorescent staining is seen in the cytoplasm of hepatocytes **(A)**, and especially in perinuclear areas **(B)**. No nuclear staining is demonstrable. Thymic stromal lymphopoietin (TSLP) immunostaining is detected in inflammatory infiltrates of portal tracts **(C)** and in biliary epithelium **(D, E)**. The fluorescence signal is completely abolished by preincubation of the anti-TSLP antibody with TSLP protein **(F)**. FITC, fluorescein isothiocyanate.

TSLP-specific immune reactive material was demonstrated in 31 of 36 (86%) and 19 of 24 (79%) liver tissue specimens from CV and non-CV patients, respectively. As shown in Figure 1, although TSLP was detected in the cytoplasm of hepatocytes, the number of stainable liver cells varied greatly among liver specimens: TSLP was in fact randomly distributed throughout the liver sections but without an obvious topographical relationship to liver structures. No distinct nuclear staining pattern was noted. Positivity of cell membranes or submembranes was infrequent (Figure 1A,B). In addition, TSLP immune deposits were detected in portal tracts containing inflammatory infiltrates (Figure 1C) and in biliary epithelium (Figure 1D,E).

**Figure 2:**
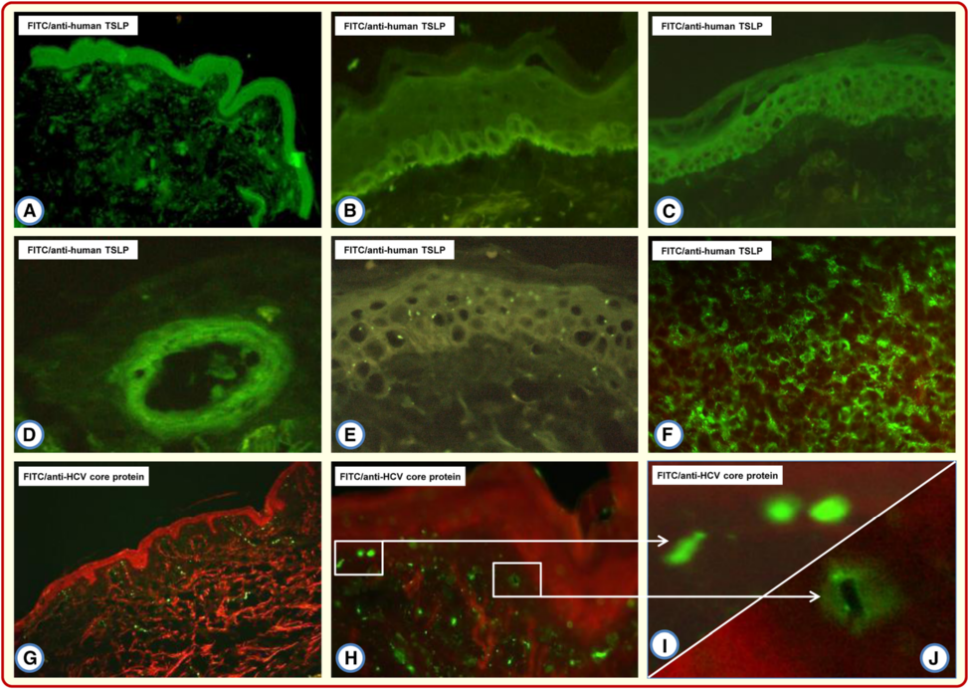
**Detection of thymic stromal lymphopoietin and hepatitis C virus core proteins in the skin biopsies of cryoglobulinemic vasculitis patients by indirect immunofluorescence.** Thymic stromal lymphopoietin (TSLP) immunoreactivity is present in the epidermis **(A)**, both in basal **(B)** and suprabasal **(C)** layers. In the dermis, TSLP immune reactants are localized in the vessel walls **(D)**. There is no TSLP signal in the skin of normal subjects **(E)**. Intense signal of TSLP-specific immune reactants in human tonsil section as positive control **(F)**. Hepatitis C virus (HCV) core protein immune deposits are found only in the dermis, whereas keratinocytes never displayed positivity **(G)**. Intravascular HCV core immunostaining completely fills the vessel lumen **(H, I)** or is localized perivascularly **(J)**. FITC, fluorescein isothiocyanate.

TSLP and HCV core proteins were studied in skin biopsy samples from all CV and non-CV patients and from healthy controls. The results showed that TSLP protein was mostly restricted to the basal and suprabasal layers of the epidermis (Figure 2A,B,C) in the large majority of both CV patients (27/31, 87%) and non-CV patients (15/19, 79%). No peculiar features were detected in the remaining TSLP distribution patterns. Analysis of the dermal layer showed TSLP protein deposition mainly in blood vessel walls (Figure 2D). Similarly, HCV core protein could be demonstrated within and/or around dermal vessels. Intravascular core reactivity appeared as coarse deposits completely filling the vessel lumen or as immune reactants within vessel walls and in the perivascular area (Figure 2G,H,I,J). Attempts to detect deposits of HCV-encoded core protein in keratinocytes consistently met with failure. None of the skin biopsy tissues from non-CV patients or healthy individuals were positive for TSLP or HCV core protein.

### Thymic stromal lymphopoietin mRNA and HCV RNA amplicons in the skin

Notably, although TSLP mRNA expression was demonstrated in the skin samples of CV and non-CV patients and in those of healthy controls, HCV RNA amplification products were present in only two (6%) of 36 skin specimens from CV patients and in none of those from either the non-CV patients or healthy individuals.

The relationship between TSLP protein, specific mRNA expression, and the occurrence of HCV core protein or viral RNA is summarized in Table 2. TSLP protein was upregulated and revealed by immunofluorescence in 33 (92%) CV patients and in five (21%) non-CV patients (*P* <0.0001), but in none of the controls (*P* <0.0001). HCV core protein was detected in the skin tissue of 29 (81%) CV patients and two (8%) non-CV patients (*P* <0.0001). Conversely, all of the skin biopsies from healthy subjects tested negative for the immune detection of both TSLP protein and HCV core protein.

**Table 2:** **Thymic stromal lymphopoietin protein and gene expression in relation to HCV core protein and HCV RNA detection in skin tissue from chronically HCV-infected patients with and without cryoglobulinemic vasculitis and from healthy controls**

	**Skin tissue**				
**Category**	**TSLP**			**HCV**	
	**Protein**	**mRNA**	**(%)**	**Core protein**	**RNA**
Cryoglobulinemic patients (*n* = 36)	33 (92)	36 (100)		29 (81)	2 (6)
	*P* <0.0001			*P* <0.0001	
Noncryoglobulinemic patients (*n* = 24)	5 (21)	24 (100)		2 (8)	0
	NS			NS	
Healthy controls (*n* = 10)	0	10 (100)		0	0

HCV, hepatitis C virus; NS, not significant; TSLP, thymic stromal lymphopoietin.

### Thymic stromal lymphopoietin gene quantitation in liver and skin compartments

**Figure 3:**
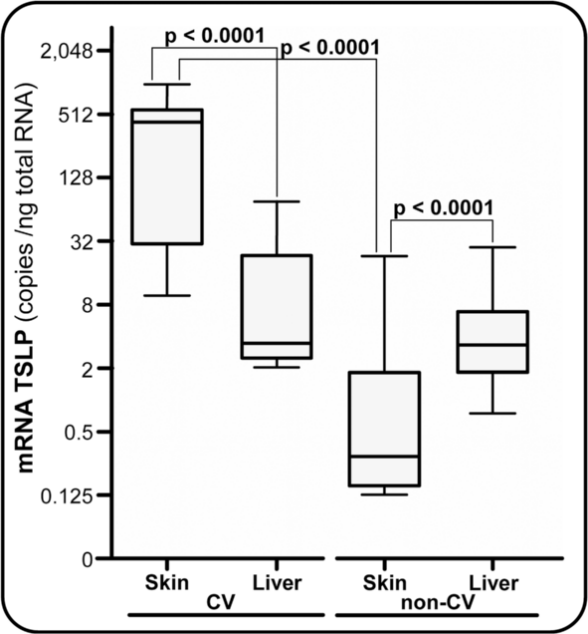
**Thymic stromal lymphopoietin gene expression measurement.** Absolute quantitative real-time RT-PCR in the skin and liver tissues of cryoglobulinemic vasculitis (CV) and non-CV patients. TSLP, thymic stromal lymphopoietin.

Potential differences in TSLP gene regulation between specific biologic compartments were searched for by measuring mRNA expression levels in the skin and in the corresponding liver tissue samples of CV and non-CV patients (Figure 3). A median of 434 (range, 10 to 1,026) mRNA copies/ng total RNA was estimated in the skin and 3.4 (range, 2.1 to 74) mRNA copies/ng total RNA in the liver samples (*P* <0.0001) of CV patients. In sharp contrast, TSLP gene expression was significantly downregulated in the skin compared with the liver in non-CV patients, with a median of 0.29 (range, 0.13 to 22.2) mRNA copies/ng total RNA and 3.3 (range, 0.75 to 28.4) mRNA copies/ng total RNA (*P* <0.0001), respectively. TSLP gene expression was thus strikingly different in the skin from CV patients compared with that from non-CV patients (*P* <0.0001).

### Serum TSLP soluble protein

Basal serum levels of TSLP protein were measured in all HCV-positive CV and non-CV patients and in all healthy subjects. At the time of serum TSLP measurement, none of the HCV-infected patients was receiving specific antiviral treatment and/or immunosuppressive drugs. TSLP levels were significantly higher in CV patients (median, 186 pg/ml; range, 97 to 435) than in either non-CV patients (median, 70.7 pg/ml; range, 16 to 157) or healthy subjects (median, 0 pg/ml; range, 0 to 18) (*P* <0.0001 in both cases).

Spearman’s rank correlation test was used to assess the strength of the association between basal TSLP concentrations and virologic and laboratory parameters (Table 3). A positive correlation was found with serum HCV nonenveloped core protein (*P* = 0.02), C1q (*P* = 0.003), C3 (*P* = 0.0001), and number of peripheral CD20^+^ cells (*P* = 0.0009). On the contrary, there was no relationship with mean age of the patients, circulating HCV RNA or cryoprotein levels, rheumatoid factor, serum concentrations of IgM and C4 complement component, and alanine aminotransferase activity.

**Table 3:** **Spearman’s rank correlation analysis of serum thymic stromal lymphopoietin levels versus laboratory/clinical parameters in 36 patients with HCV-related cryoglobulinemic vasculitis**

**Parameter**	***r*** **coefficient**	**95% confidence interval**	***P*** **value** ^**a**^
Epidemiology			
Age (years)	−0.2144	−0.5147 to 0.1328	0.2092
Virology			
Serum HCV RNA (log IU/ml)	−0.2000	−0.5307 to 0.1836	0.2893
Serum HCV nonenveloped core protein (pg/ml)	0.4182	0.0768 to 0.6718	0.02
Laboratory			
Cryocrit (%)	−0.0845	−0.4104 to 0.2605	0.6241
Rheumatoid factor (IU/ml)	−0.1182	−0.4520 to 0.2447	0.5125
IgM (mg/dl)	0.1000	−0.2619 to 0.4373	0.5798
C1q (mg/dl)	0.6571	0.2605 to 0.8640	0.0030
C3 (mg/dl)	0.7335	0.5137 to 0.8629	0.0001
C4 (mg/dl)	0.1388	−0.2085 to 0.4551	0.4194
ALT (IU/l)	0.1763	−0.2072 to 0.5129	0.3514
Peripheral CD20^+^ cells (%)	0.7143	0.3581 to 0.8890	0.0009

HCV, hepatitis C virus. ^a^ *P* <0.05 was defined as significant.

### Therapy-induced modifications of serum TSLP levels

**Figure 4:**
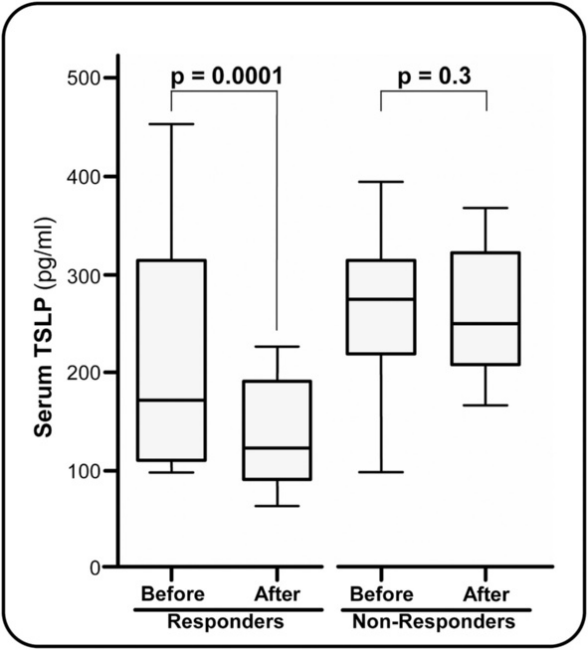
**Serum levels of soluble thymic stromal lymphopoietin protein in cryoglobulinemic vasculitis patients responsive and unresponsive to antiviral therapy.** TSLP, thymic stromal lymphopoietin.

Changes in the serum TSLP levels were investigated following therapy in all CV patients. Baseline serum levels of this cytokine were slightly but not significantly lower in 16 responder patients (44.4%) compared with 20 nonresponder patients (55.6%). In the first group, the administration of pIFNα/RBV combination therapy resulted in a dramatic improvement of cryoglobulin-related signs and symptoms, a remarkable decrement of the cryocrit, and the disappearance of serum HCV RNA. This complete response was found to be associated with a significant drop in TSLP serum levels (Figure 4). In sharp contrast, TSLP levels remained roughly unchanged in the group of unresponsive patients.

## Discussion

The main features of this study can be summarized as follows: untreated patients with HCV-related CV exhibit a significant median increase in the TSLP mRNA transcripts of skin mRNA tissue samples compared with the corresponding liver samples; basal and suprabasal keratinocytes and hepatocytes have been identified as the sources of TSLP; serum TSLP protein levels are significantly higher in CV patients than in either non-CV patients or healthy subjects; higher serum levels of TSLP in CV patients are associated with a higher frequency of circulating monoclonal/oligoclonal expansions of B cells in the large majority of these patients; and achievement of complete response by the combined pIFNα/RBV therapy results in a highly significant reduction of serum TSLP.

One obvious question stemming from our study is whether the correlations reported above indeed reflect a specific mechanistic role of TSLP in the onset of CV or are simply the result of generalized immune activation in this condition. Several observations, however, argue against a simplistic interpretation. We have already mentioned in the Introduction that TSLP transgenic mice often develop mixed cryoglobulins and cryoglobulinemic type I membrano-proliferative glomerulonephritis as well as additional features that closely resemble important aspects of the human disease [7]. In addition, a growing number of studies, highlighted in two recent reviews [20,21], have provided insightful advances into TSLP’s pleomorphic roles in diverse disease conditions, including allergy, autoimmunity, and cancer, thus making it unlikely that the elevated TSLP levels are the consequence of rather than the driver of CV.

The changes within the skin microenvironment that induce TSLP gene expression and the upregulation of TSLP protein are as yet unclear, given that in the keratinocytes of HCV-infected non-CV patients TSLP gene expression was downregulated and TSLP protein production was absent. It seems unlikely that the upregulation of TSLP in the skin of CV patients is directly dependent on HCV, in that we have never been able to convincingly demonstrate HCV RNA amplicons in skin tissue samples and have no evidence of a direct relationship between HCV RNA levels and the type of and/or the extent of skin damage.

By analogy with the results obtained *in vitro* by Lee and colleagues [14], we have shown *in vivo* that chronic HCV infection is able to induce TSLP gene expression and the hepatocyte production of the protein, which is then released in the circulation. In addition, circulating cold-precipitable immune complexes are crucial to the pathogenesis of cryoglobulin-related cutaneous vasculitis [11], characterized by activation of the complement pathway, with subsequent damage to endothelial cells and the initiation of a local inflammatory response [12,22].

TSLP protein was highly expressed in skin biopsies from CV patients, forming large deposits in dermal vessel walls, a finding reminiscent of the TSLP deposits in the skin of patients with diffuse cutaneous systemic sclerosis [23]. It can be assumed that HCV core protein affects the pathobiology of T-cell responses by inducing anergy-related genes [24] and *in loco* generation of a T-helper 17 response [25]. This raises the intriguing possibility of a cross-talk between hepatocyte-derived TSLP and skin tissue damage, resulting in the polarization of DCs and CD4^+^ T-cell differentiation [1].

In response to pathogens, DCs both produce and respond to TSLP, thereby creating a potential autocrine loop [26]. According to this view, DCs can be instructed by TSLP produced in inflamed tissues to migrate into the interstitial environment of peripheral tissues and to initiate a suitable immune response [27]. Notably, because of the critical role of nuclear factor-κB downstream of the toll-like receptor signaling pathway, it is not surprising that toll-like receptor agonists as well as infectious agents are effective inducers of TSLP expression in epithelial cells [28].

Although TSLP expression can be induced by the ongoing HCV infection of hepatocytes, we failed to demonstrate a direct relationship between systemic levels of TSLP and circulating viral RNA. Conversely, serum TSLP levels correlated well with those of HCV core protein, indicating an association between higher amounts of the former and HCV RNA-free structures.

An important point of our study is the detection of a significant reduction of TSLP serum levels in patients responsive to pIFNα/RBV combination therapy, probably reflecting the appropriate blockade of TSLP release by hepatocytes and keratinocytes. Conversely, the unchanged levels in unresponsive patients suggest persisting HCV-mediated induction of TSLP production that contributes to immune system activation. Although the addition of the anti-CD20 monoclonal antibody rituximab to the pIFNα/RBV combination has been shown to result in higher long-term response rates [29], rituximab alone does not seem to affect the serum TSLP levels: in a pilot study carried out in nine CV patients different from those enrolled in this study, no significant variations of serum TSLP levels were detected following the administration of four weekly intravenous infusions of 375 mg/m^2^ rituximab monotherapy (data not shown).

In a proof-of-concept study, the administration of a fully human anti-TSLP monoclonal antibody G2λ to a small cohort of patients with stable allergic asthma resulted in a significant attenuation of allergy-induced early and late asthmatic responses [30]. No therapeutic proof-of-principle attempts with anti-TSLP monoclonal antibodies have so far been reported in CV patients, but it seems reasonable to hypothesize that TSLP blockade is a potential, targeted therapeutic strategy that could be added to the current therapies of CV [31].

## Conclusions

This study points out a novel biological role for TSLP. Cutaneous expression of TSLP by the keratinocytes of patients with HCV-related CV may be pivotal in perpetuating the activation of tissue-resident immunocytes and in attracting inflammatory cells, thus creating an environment favorable to the onset of vasculitis. The local production of TSLP by hepatocytes as well as keratinocytes causes a significant increase in its systemic concentrations, a step sufficient to promote a break in B-cell homeostasis and likely to induce B-lineage-dependent autoimmunity.

Further studies are necessary to evaluate the molecular basis underlying TSLP upregulation and B-cell derangement in patients with HCV-related CV. Although a deeper comprehension of cell-specific signaling pathways awaits to be defined, given that TSLP has been found capable of directly acting on CD4^+^ T cells and of promoting proliferation and T-helper 2 differentiation of naïve CD4^+^ T cells through the induction of interleukin-4 [20], it seems reasonable to hypothesize that the overexpression of mRNA TSLP in the skin of CV patients and the consequent cytokine milieu play an important role in the inflammatory responses that characterize this peculiar type of HCV-induced vasculitis.

## Abbreviations

CV: cryoglobulinemic vasculitis
DC: dendritic cell
HCV: hepatitis C virus
IFNα: interferon alpha
RBV: ribavirin
TSLP: thymic stromal lymphopoietin
